# Individualized Prediction of Survival Benefits of Pancreatectomy Plus Chemotherapy in Patients With Simultaneous Metastatic Pancreatic Cancer

**DOI:** 10.3389/fonc.2021.719253

**Published:** 2021-09-16

**Authors:** Duorui Nie, Guihua Lai, Guilin An, Zhuojun Wu, Shujun Lei, Jing Li, Jianxiong Cao

**Affiliations:** ^1^Graduate School, Hunan University of Chinese Medicine, Changsha, China; ^2^School of Traditional Chinese Medicine, Ningxia Medical University, Yinchuan, China; ^3^Department of Oncology, The First Hospital of Hunan University of Chinese Medicine, Changsha, China; ^4^School of Continuing Education, Hunan University of Chinese Medicine, Changsha, China

**Keywords:** metastatic pancreatic cancer, surgery, chemotherapy, prognosis analysis, nomogram

## Abstract

**Background:**

Metastatic pancreatic cancer (mPC) is a highly lethal malignancy with poorer survival. However, chemotherapy alone was unable to maintain long‐term survival. This study aimed to evaluate the individualized survival benefits of pancreatectomy plus chemotherapy (PCT) for mPC.

**Methods:**

A total of 4546 patients with mPC from 2004 to 2015 were retrieved from the Surveillance, Epidemiology, and End Results database. The survival curve was calculated using the Kaplan-Meier method and differences in survival curves were tested using log-rank tests. Cox proportional hazards regression analyses were performed to evaluate the prognostic value of involved variables. A new nomogram was constructed to predict overall survival based on independent prognosis factors. The performance of the nomogram was measured by concordance index, calibration plot, and area under the receiver operating characteristic curve.

**Results:**

Compared to pancreatectomy or chemotherapy alone, PCT can significantly improve the prognosis of patients with mPC. In addition, patients with well/moderately differentiated tumors, age ≤66 years, tumor size ≤42 mm, or female patients were more likely to benefit from PCT. Multivariate analysis showed that age at diagnosis, sex, marital status, grade, tumor size, and treatment were independent prognostic factors. The established nomogram has a good ability to distinguish and calibrating.

**Conclusion:**

PCT can prolong survival in some patients with mPC. Our nomogram can individualize predict OS of pancreatectomy combined with chemotherapy in patients with concurrent mPC.

## Background

Pancreatic cancer (PC) is a highly lethal malignancy, known as the “king of cancers”. It was reported to cause 432,242 deaths worldwide in 2018, ranking fourth among cancer-related deaths (1). By 2030, it will be the second leading cause of cancer-related deaths (2). The poor prognosis for PC is associated with a later stage of diagnosis. It is reported that approximately 50% of patients are newly diagnosed with metastatic pancreatic cancer (mPC) (3). Moreover, the aggressive biological behavior of pancreatic cancer causes most patients who receive pancreatic cancer at an early stage to experience recurrence and metastasis (4). Therefore, the management of mPC deserves more attention. However, the treatment options for patients with mPC are limited, and systemic chemotherapy with Leucovorin, fluorouracil, irinotecan, and oxaliplatin or gemcitabine plus Nab-paclitaxel was recommended as the first-line treatment (5). Although significantly longer survival than gemcitabine monotherapy, the overall survival (OS) was only improved by a few months, and the clinical benefit was still limited (6, 7).

Surgery is the only cure for pancreatic cancer, but it is still underused in patients with early-stage pancreatic cancer because of concerns about its safety and complications (8). It is generally believed that metastatic disease is a contraindication to resection, but in the absence of effective treatment, the survival benefits of patients with mPC undergoing surgical resection are of concern (9–12). And with the advancement of surgical techniques and systemic chemotherapy, the perioperative mortality of patients with pancreatic cancer has dropped to 3%, and the 5-year survival rate has increased to about 30-40% (13). Pancreatectomy is considered to be a safe and effective treatment, but most patients who undergo surgery, even those who undergo radical resection, will eventually have a recurrence of the disease (14). Hence, the combination of chemotherapy seems to be a new combination therapy that offers hope for the treatment of pancreatic cancer. Highly selected patients with mPC may benefit from pancreatectomy and chemotherapy (15–17). But because they are small, single-center retrospective studies, we cannot draw reliable conclusions from these studies. Therefore, the exact role of pancreatectomy combined with chemotherapy deserves a more systematic evaluation.

Therefore, this study evaluated the prognostic effect of pancreatectomy combined with chemotherapy in patients with mPC. In addition, we have also explored the prognostic factors that affect mPC and established a nomogram to manage this type of patient.

## Methods

### Patient Population

The Surveillance Epidemiology and End Results (SEER) database collects tumor clinicopathological information from 18 population-based cancer registries covering nearly 27.8% of the U.S. population, gathering information on patient demographics, primary tumor site, tumor type stage at diagnosis, the first course of treatment, and follow-up patients’ vital status. The SEER database has limited access, and we have obtained SEER licenses (login number: 10952-Nov2019) to access the research data.

**Figure 1:**
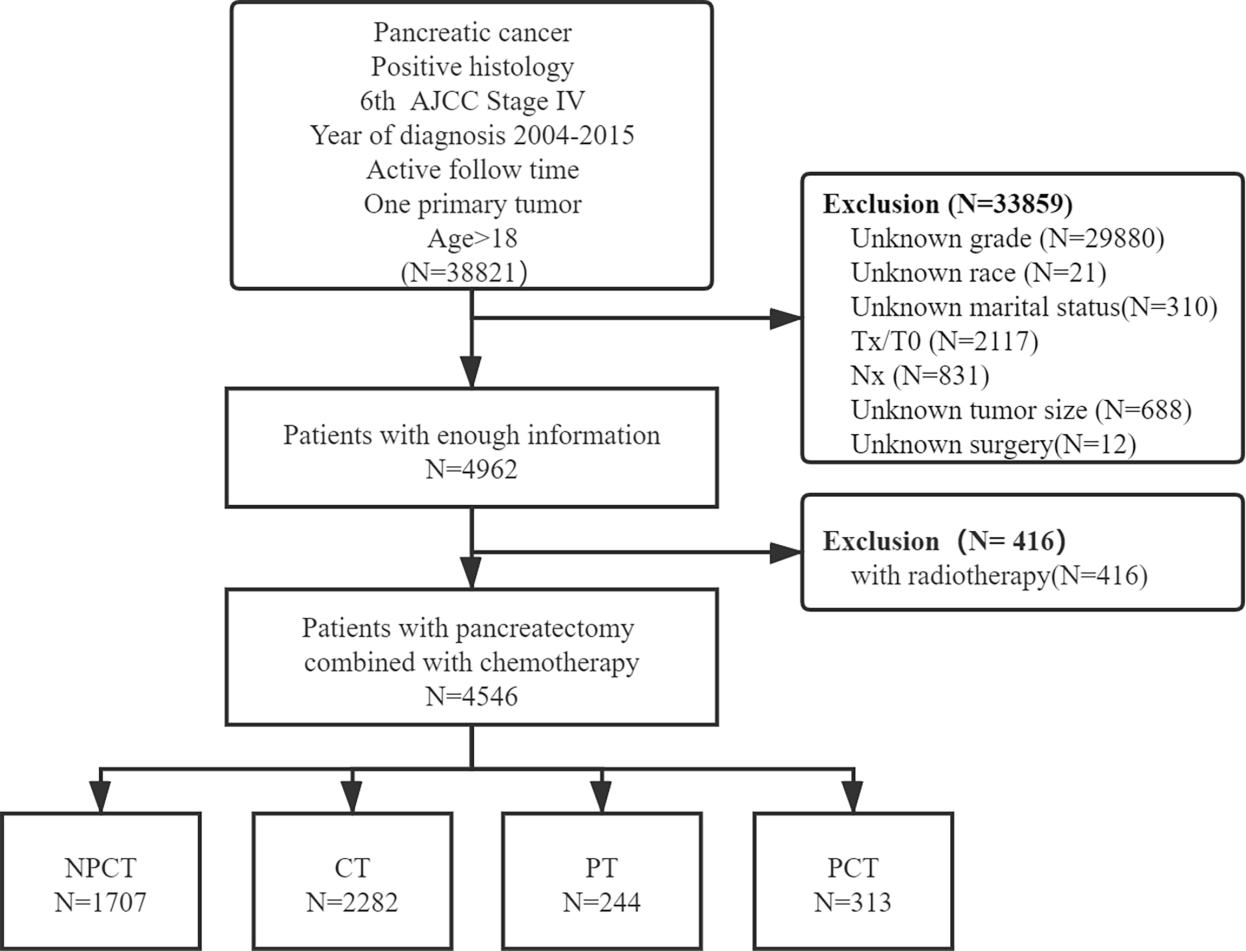
Flowchart of patient selection for this study.

Patients with simultaneous metastatic pancreatic cancer were retrieved from 18 registries of the SEER Program (1975-2016), which was submitted in November 2018, by using SEER*Stat 8.38 software (18). Patients meeting the following criteria were included: (1) the patient was diagnosed with the International Classification of Diseases for Oncology, Third Edition (ICD-O-3, histology code: 8000/3: Neoplasm, malignant, 8010/3: Carcinoma, NOS, 8070/3: Squamous cell carcinoma, NOS, 8140/3: Adenocarcinoma, NOS, 8480/3: Mucinous adenocarcinoma, 8481/3: Mucin-producing adenocarcinoma, 8490/3: Signet ring cell carcinoma, 8500/3: Infiltrating duct carcinoma, NOS, 8560/3: Adenosquamous carcinoma; and the ICD-O-3 site code: C25.0-C25.9); (2) diagnosis was made between 2004 and 2015, (3) had 6^th^ American Joint Committee on Cancer (AJCC) staging system M1 disease (4) age at diagnosis ≥18; (5) diagnosed with positive histology or cytology; (6) only one primary tumor; (7) with active follow-up time. And the following patients were excluded: (1) unknown clinical information, including T stage, N stage, race, grade, marital status, tumor size, surgery; (2) had radiotherapy. A detailed flow chart of patient screening is shown in **Figure 1**.

### Covariates and Endpoint

The following variables were included in the study: gender, age at diagnosis, race, primary site, year of diagnosis, marital status at diagnosis, grade, tumor size, AJCC stage, radiotherapy, chemotherapy, primary site surgery, survival months, and vital status. For the purposes of statistical analysis, those patients whose marital status was widowed, separated, divorced, or single (a domestic partner, or never married) were classified as “unmarried”. The tumor is located in “C25.3-Pancreatic Duct”, “C25.7-Other specified parts of Pancreas”, “C25.4-Islets of Langerhans”, “C25.9-Pancreas, NOS” were classified as “Others”. Consequently, the primary sites were categorized as “Head”, “Body”, “Tail”, “Others”, “Overlapping lesion”. According to the code of surgery and chemotherapy, the treatment is divided into four categories: patients who did not receive pancreatectomy or chemotherapy (NPCT), patients who received chemotherapy merely (CT), patients receiving pancreatectomy only (PT), and patients who received pancreatectomy and chemotherapy (PCT). Since “tumor size” and “age at diagnosis” were quantifiable data, we converted them into categorical variables based on the median of the overall cohort. The endpoint event for this study is OS, which is defined as the time from the date of initial treatment to the patient’s death of any cause or the most recent follow-up.

### Statistical Analysis

Descriptive statistics were performed for patients’ demographic and tumor characteristics. The comparison of the categorical variable among multiple groups were measured by Chi-square tests or Fisher’s exact test, while continuous variable groups were tested for Kruskal - Wallis test. The survival curve was calculated using the Kaplan-Meier method and differences in survival curves were tested using log-rank tests. Cox proportional hazards models were used to evaluate variables that have independent predictive effects on the OS. Only variables that were significantly associated with OS in the univariate Cox analysis were included in the multivariate Cox analysis. Hazard ratios (HRs) and 95% confidence intervals (CIs) were also estimated using Cox proportional hazards models. All patients are used to form a training set to assess the prognostic role of surgery and chemotherapy, perform cox analysis and develop the nomogram.

Based on the results of the multivariate Cox proportional hazards model, the nomogram with 6-, 12- 18- month survival rates were plotted. We evaluated the performance of the nomogram by discrimination and calibration (19). Discrimination is the ability of the model to correctly distinguish between non-events and events, and is quantified by Harrell’s consistency index (C-index) and time-dependent receiver operating characteristic (tROC) curve. Calibration compares the difference between the predicted probability and the actual survival rate and is represented by a calibration plot. The bootstrap analyses with 1000 resample were used to calculate C-indexes and generate calibration plots for internal validation of the model (20). All statistical tests were performed using SPSS Statistics 26.0 software (IBM SPSS, Inc., Chicago, IL, USA) and R 4.0.4 (http://www.r-project.org/). The statistical test was two-sided and P <0.05 was considered statistically significant.

## Results

### Patient Characteristic

A total of 4,546 patients were enrolled in our study. Among them, 313 patients with “PCT”, 244 patients with “PT”, 2282 patients with “CT”, 1707 patients with “NPCT”. **Table 1** shows the patients’ clinicopathological characteristics with different therapeutic modalities. The median age was 66 years (range 58-74), with 2466 (54.2%) males and 2080 (45.8%) females. Poorly differentiated was the most common grade for mPC (n=2415, 53.1%), followed by moderately differentiated (n=1649, 36.3%), well-differentiated (n=372, 8.2%) and undifferentiated (n=110, 2.4%). Chi-square test showed significant differences in some variables and treatment patterns, including age at diagnosis, year of diagnosis, race, tumor size, marital status, primary site, T stage, N stage (*P<* 0.01).

**Table 1:** Clinicopathological Characteristics of mPC patients with PCT, PT, CT or with no treatment.

Variable	Level	Overall (N = 4546)	NPCT (N = 1707)	CT (N = 2282)	PT (N = 244)	PCT (N = 313)	P-value
**Age at diagnosis (median [IQR])**		66.0 [58.0, 74.0]	69.0 [60.0, 78.0]	65.0 [57.0, 72.0]	67.5 [58.8, 76.0]	63.0 [56.0, 70.0]	**<0.001**
**Age at diagnosis (%)**	≤66 years	2328 (51.2)	720 (42.2)	1293 (56.7)	119 (48.8)	196 (62.6)	**<0.001**
	>66 years	2218 (48.8)	987 (57.8)	989 (43.3)	125 (51.2)	117 (37.4)	
**Sex (%)**	Female	2080 (45.8)	792 (46.4)	1014 (44.4)	125 (51.2)	149 (47.6)	0.153
	Male	2466 (54.2)	915 (53.6)	1268 (55.6)	119 (48.8)	164 (52.4)	
**Grade (%)**	Well	372 (8.2)	116 (6.8)	204 (8.9)	24 (9.8)	28 (8.9)	**<0.001**
	Moderately	1649 (36.3)	558 (32.7)	835 (36.6)	112 (45.9)	144 (46.0)	
	Poorly	2415 (53.1)	993 (58.2)	1184 (51.9)	100 (41.0)	138 (44.1)	
	Undifferentiated	110 (2.4)	40 (2.3)	59 (2.6)	8 (3.3)	3 (1.0)	
**Year of diagnosis (%)**	2004-2009	1994 (43.9)	805 (47.2)	921 (40.4)	132 (54.1)	136 (43.5)	**<0.001**
	2010-2015	2552 (56.1)	902 (52.8)	1361 (59.6)	112 (45.9)	177 (56.5)	
**Race (%)**	Black	632 (13.9)	284 (16.6)	291 (12.8)	30 (12.3)	27 (8.6)	**<0.001**
	Other	362 (8.0)	134 (7.9)	177 (7.8)	19 (7.8)	32 (10.2)	
	White	3552 (78.1)	1289 (75.5)	1814 (79.5)	195 (79.9)	254 (81.2)	
**Tumor size (median [IQR])**		42.0 [31.0, 56.0]	43.0 [31.0, 58.0]	42.0 [32.0, 55.0]	40.0 [30.0, 60.0]	38.0 [28.0, 51.0]	**<0.001**
**Tumor size (%)**	≤42 mm	2309 (50.8)	835 (48.9)	1148 (50.3)	129 (52.9)	197 (62.9)	**<0.001**
	>42 mm	2237 (49.2)	872 (51.1)	1134 (49.7)	115 (47.1)	116 (37.1)	
**Marital status at diagnosis (%)**	Married	2750 (60.5)	878 (51.4)	1483 (65.0)	164 (67.2)	225 (71.9)	
	Unmarried	1796 (39.5)	829 (48.6)	799 (35.0)	80 (32.8)	88 (28.1)	
**Primary Site (%)**	Body	770 (16.9)	275 (16.1)	448 (19.6)	16 (6.6)	31 (9.9)	**<0.001**
	Head	2003 (44.1)	751 (44.0)	954 (41.8)	135 (55.3)	163 (52.1)	
	Others	368 (8.1)	167 (9.8)	169 (7.4)	15 (6.1)	17 (5.4)	
	Overlapping	480 (10.6)	170 (10.0)	263 (11.5)	20 (8.2)	27 (8.6)	
	Tail	925 (20.3)	344 (20.2)	448 (19.6)	58 (23.8)	75 (24.0)	**<0.001**
**T stage (%)**	T1	141 (3.1)	69 (4.0)	64 (2.8)	6 (2.5)	2 (0.6)	
	T2	1393 (30.6)	586 (34.3)	746 (32.7)	27 (11.1)	34 (10.9)	
	T3	1883 (41.4)	639 (37.4)	832 (36.5)	170 (69.7)	242 (77.3)	
	T4	1129 (24.8)	413 (24.2)	640 (28.0)	41 (16.8)	35 (11.2)	
**N stage (%)**	N0	2527 (55.6)	1034 (60.6)	1338 (58.6)	68 (27.9)	87 (27.8)	**<0.001**
	N1	2019 (44.4)	673 (39.4)	944 (41.4)	176 (72.1)	226 (72.2)	

Statistically significant inter-group comparisons of the four treatments are shown in bold (P < 0.05).

### Prognosis Analysis

**Figure 2:**
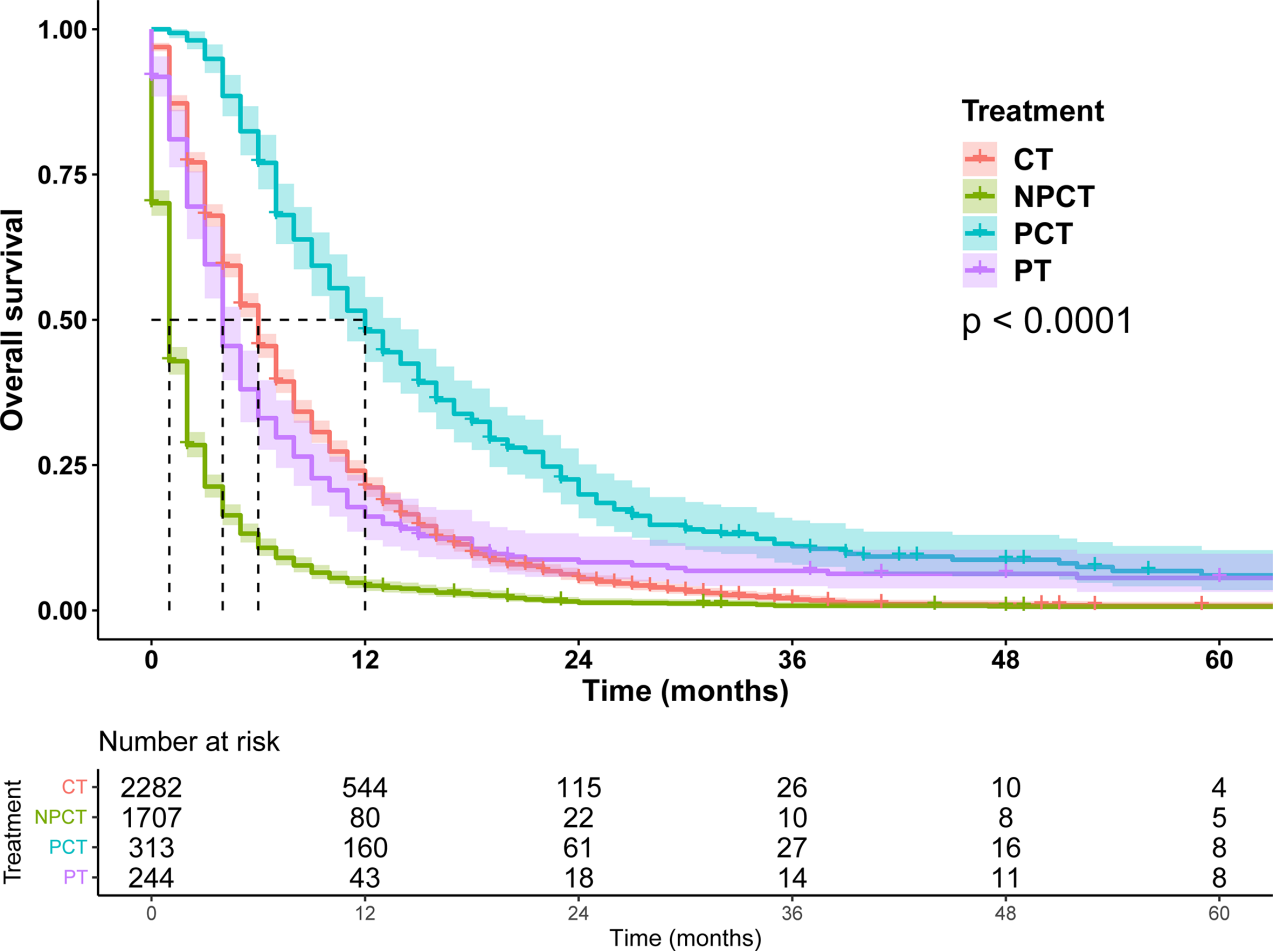
Survival curves for patients with metastatic pancreatic cancer in different treatment modalities.

Due to poor prognosis, the median follow-up time was 4 months (range, 0-150 months). To investigate the prognostic role of pancreatectomy and chemotherapy in mPC, we performed survival analysis, and survival curves were shown in **Figure 2**. The results show that pancreatectomy combined with chemotherapy can significantly improve the prognosis of patients with mPC compared to pancreatectomy or chemotherapy alone (*P<*0.001). The median OS for patients with mPC receiving PCT was 12 months, while 6 months for CT, 4 months for PT, and 1 month for NPCT.

### Subgroup Analysis

**Figure 3:**
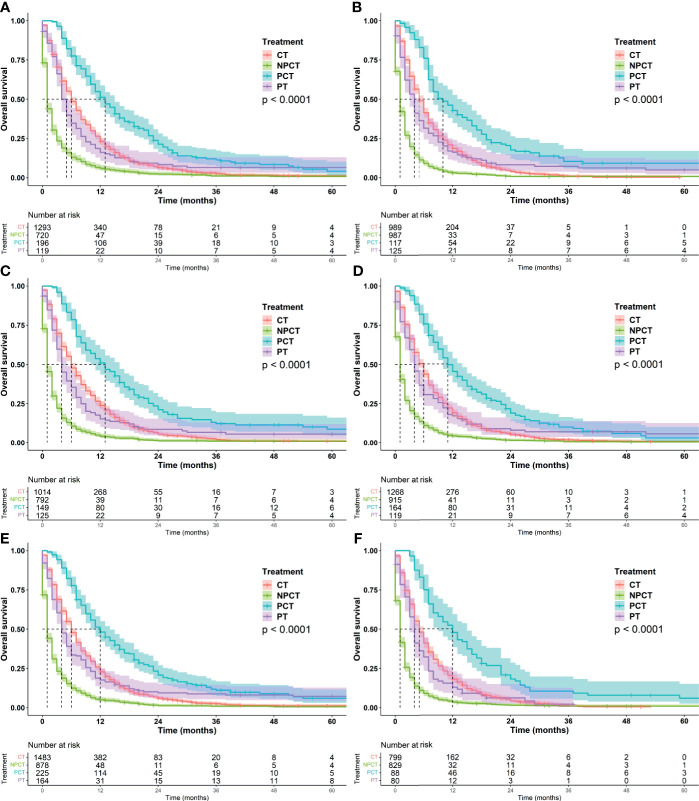
Kaplan-Meier survival curves for subgroup analysis of patients with metastatic pancreatic cancer with different clinical characteristics. **(A)**: age ≤ 66 years, **(B)**: age>66 years, **(C)**: female, **(D)**: male, **(E)**: married, **(F)**: unmarried.

**Figure 4:**
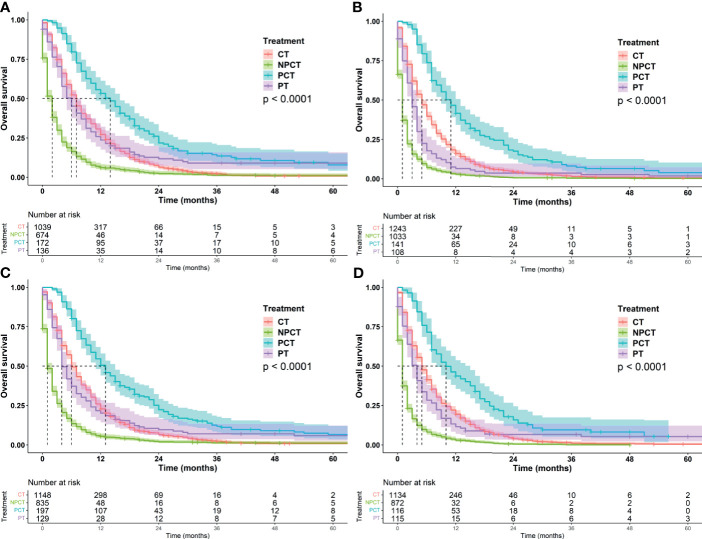
Kaplan-Meier survival curves for subgroup analysis of patients with metastatic pancreatic cancer with different tumor characteristics. **(A)** grade: well/moderately differentiated **(B)** grade: Poor/Undifferentiated, **(C)**: tumor size ≤ 42mm, **(D)**: tumor size>42mm.

Although pancreatectomy combined with chemotherapy has a significant benefit in the overall population, it is not clear whether there is a benefit in the characteristic population, so we conducted exploratory stratification, such as age at diagnosis, sex, marital status, tumor size, and histological grade. We found that pancreatectomy combined with chemotherapy can significantly prolonged OS time in patients with mPC, regardless of age at diagnosis, sex, marital status, tumor size, and histological grade (**Figures 3** and **4**).

It was further found that among patients with age ≤66 years and tumor ≤42 mm, patients receiving PCT had a more significant benefit, with a median OS of 13 months (95%CI: 11-15). Patients with well/moderately differentiated tumors or females also had a greater survival benefit, with a median OS of 14 months (95%CI: 11-16). We speculate that these may be favorable populations for surgery and chemotherapy

### Construction and Validation of a Nomogram

To further investigate the risk factors for long-term survival of mPC, univariate and multivariate Cox regression analyses were used to identify independent prognostic factors (**Table 2**). Multivariate Cox regression results also indicated that PCT (HR = 0.250, 95% CI: 0.219-0.285, *P<*0.001) were a favorable prognostic factor for mPC. In addition, age at diagnosis, tumor size, marital status at diagnosis, sex, grade were also independent predictive factors (*P< 0*.001).

**Table 2:** Univariate and multivariate analysis for mPC patients.

Variable	Levels	Univariate		Multivariate	
		HR (95%CI)	P-value	HR (95%CI)	P-value
**Age at diagnosis**	≤66	Reference		Reference	
	>66	1.275 (1.202-1.354)	**<0.001**	1.218 (1.146-1.295)	**<0.001**
**Gender**	Female	Reference		Reference	
	Male	1.079 (1.016-1.145)	**0.013**	1.105 (1.039-1.176)	**0.001**
**Race**	Black	Reference		Reference	
	Other	0.863 (0.756-0.985)	**0.029**	0.961 (0.841-1.099)	0.565
	White	0.866 (0.794-0.944)	**0.001**	0.927 (0.848-1.013)	0.093
**Material status at diagnosis**	Married	Reference		Reference	
	Unmarried	1.267 (1.193-1.347)	**<0.001**	1.161 (1.089-1.237)	**<0.001**
**Tumor size**	≤42	Reference		Reference	
	>42	1.242 (1.17-1.318)	**<0.001**	1.238 (1.164-1.317)	**<0.001**
**Location**	Body	Reference			
	Head	0.936 (0.860-1.018)	0.123	–	
	Others	1.071 (0.944-1.216)	0.284		
	Overlapping	1.049 (0.934-1.177)	0.421		
	Tail	1.056 (0.958-1.163)	0.273		
**Histological grade**	Well differentiated	Reference			
	Moderately differentiated	1.184 (1.054-1.33)	**0.005**	1.261 (1.122-1.1417)	**<0.001**
	Poorly differentiated	1.597 (1.426-1.789)	**<0.001**	1.656 (1.477-1.4857)	**<0.001**
	Undifferentiated	1.452 (1.169-1.804)	**0.001**	1.372 (1.104-1.1706)	**0.004**
**T stage**	T1	Reference			
	T2	1.213 (1.016-1.448)	**0.033**	1.176 (0.983-1.408)	0.076
	T3	0.993 (0.834-1.183)	**0.940**	1.051 (0.879-1.258)	0.584
	T4	1.123 (0.939-1.343)	**0.203**	1.094 (0.911-1.313)	0.337
**N stage**	N0	Reference			
	N1	0.943 (0.889-1.001)	0.055		–
**Treatment**	NPCT	Reference			
	CT	0.409 (0.383-0.436)	**<0.001**	0.418 (0.391-0.447)	**<0.001**
	PT	0.432 (0.376-0.497)	**<0.001**	0.483 (0.419-0.557)	**<0.001**
	PCT	0.227 (0.199-0.258)	**<0.001**	0.250 (0.219-0.285)	**<0.001**

Statistically significant independent prognostic factors are shown in bold (P < 0.05).

**Figure 5:**
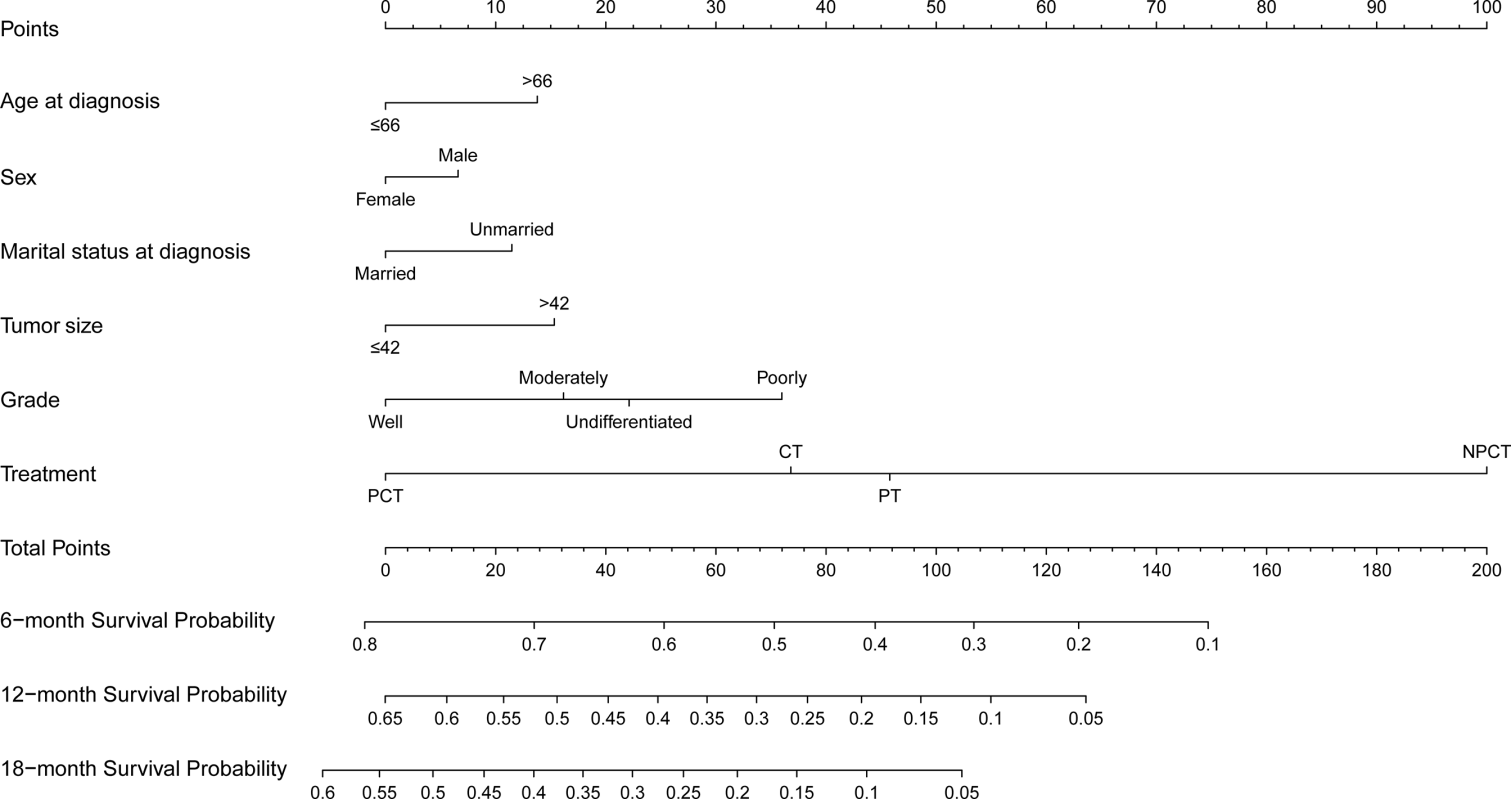
Prognosis nomogram predicting 6‐, 12‐, and 18‐month survival probability for patients with metastatic pancreatic cancer using six clinical characteristics.

**Figure 6:**
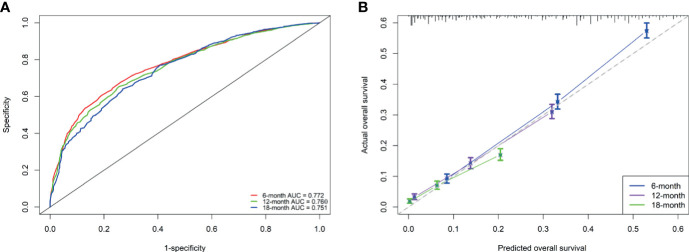
Performance of the nomogram for metastatic pancreatic cancer patients **(A, B)**. **(A)** ROC curves and AUC at 6, 12, and 18 months were used to estimate discriminating power of the nomogram. the closer the area under the curve is to 1, the better the distinguishing ability is; **(B)** Calibration curves for predicting 6-, 12-, and 18-month OS were used to estimate the prediction accuracy of the nomogram. The *x*-axis indicates the predicted overall survival probability, and the *y*-axis indicates the actual survival probability. The 45-degree line (*gray line*) indicates that the prediction agrees with actuality.

Based on the independent prognostic factors derived from multivariate Cox regression, we established a nomogram to predict 6-month, 12-month, 18-month OS probability for mPC (**Figure 5**). As shown in the nomogram, the treatment modality contributed the most to OS, followed by grade, tumor size, and age at diagnosis. The C index of our model was 0.717. After bootstrapping, it still had a good discriminative ability with a C-index of 0.716. When the tROC analysis was performed, the area under the ROC curve at 6-, 12- and 18-month was 0.772, 0.760, and 0.751, respectively (**Figure 6A**). These results all show that our model has a good discriminative ability. At the same time, the calibration analysis was performed. The calibration curve for predicting 6-, 12-, 18-month OS. was shown in **Figure 6B**, and the bootstrapping calibration plots showed the good prediction accuracy of our nomogram.

## Discussion

In this study, we analyzed the treatment data of 4546 patients with mPC, revealing meaningful treatment modalities. We found that pancreatectomy combined with chemotherapy can significantly prolong the OS of patients with mPC compared to surgery or chemotherapy alone. In addition, we provide a nomogram to estimate the OS of mPC patients, which can be used to quantify the risk factors of patients and guide clinical treatment.

It is well known that patients with mPC are prone to pain, weight loss, obstruction, and other discomforts, which seriously affect their lives. Therefore, the treatment of metastatic pancreatic cancer usually takes chemotherapy as the main treatment to delay tumor progression and increase the survival time, and symptomatic treatment including oral opioid analgesics, ethanol ablation combined with celiac plexus neurolysis by endoscopic ultrasound, nutritional support, and endoscopic biliary and duodenal stent implantation to improve the quality of life (21, 22). But these non-surgical palliative treatments were not satisfactory. Surgery, as one of the main therapies for cancer treatment, seems to offer hope for patients with metastatic pancreatic cancer whose primary tumor is resectable. In particular, two new treatment regimens released in 2011 and beyond not only improved overall survival rates for pancreatic cancer but also showed good anti-tumor activity (6, 7). Therefore, some mPC patients received surgical treatment after conversion therapy, and the other part received adjuvant chemotherapy after surgical resection, and they may achieve long-term survival (23).

A multi-center phase II clinical study (24) revealed the prognostic analysis of 33 patients receiving an intravenous and intraperitoneal infusion of paclitaxel and combined with S-1 for the treatment of peritoneal metastasis of pancreatic cancer. Among them, the median OS of 8 patients who underwent conversion surgery after neoadjuvant chemotherapy was significantly higher than that of patients without surgery (27.8 vs 14.2 months, *P* = 0.0062). This is the highest level of evidence to date for the combination of surgery and chemotherapy, revealing the possibility of long-term survival after surgery in patients with partial loss of peritoneal metastases following neoadjuvant chemotherapy. In addition, liver metastasis is the most common mode of pancreatic cancer, and it usually indicates a worse prognosis than other sites (25). Of the 535 patients with hepatic metastases from pancreatic cancer who received neoadjuvant chemotherapy, 24 patients completed chemotherapy with radiographic findings indicating hepatic metastases disappeared, normal or significantly reduced cancer antigen 19-9 expression and received pancreatic resection. The overall group had OS and progression-free survival (PFS) of 56 and 27 months, respectively (26). Despite the lack of a control group, this treatment pattern has improved a lot compared to the previous reports of the mPC bad OS and PFS.

In our study, the median OS of pancreatectomy combined with chemotherapy was 12 months. The difference in survival between our study and other studies may be due to the difference in inclusion and exclusion criteria in the cohort. Our study included patients from 2004 to 2015, patients diagnosed with stage IV pancreatic cancer before 2011 were less likely to receive the new intensive treatment regimen. But pancreatectomy combined with chemotherapy still appears to be a favorable treatment. Kim (15) collected patients diagnosed with metastatic pancreatic cancer between 2000 and 2009, 35 of whom underwent surgical resection and matched 35 unresected patients with similar tumor size and peritoneal metastasis. The results showed that pancreatectomy for stage IV pancreatic duct adenocarcinoma can significantly improve the survival rate. Postoperative chemotherapy was statistically significant for survival (HR=0.44; 95% CI:1.03-3.15; *P* = 0.003).

Although prognostic factors for survival are not equal to predictors of treatment effectiveness, these results nevertheless remind us that these prognostic factors may be useful in further selecting specific subgroups that will benefit from pancreatectomy and chemotherapy. Therefore, we established a nomogram based on independent prognostic factors to select patients who might benefit from surgery and chemotherapy for survival. Those with smaller tumors, younger age, and better histological grades, women, married, undergoing surgery and chemotherapy, seem to have long-term survival. In addition, the subgroup analysis also showed that women, smaller tumors, younger age, and better histological grade appeared to benefit more from pancreatectomy and chemotherapy. These patients are the beneficiaries of pancreatectomy and chemotherapy, probably because they can tolerate the intense treatment and their tumors are more sensitive to chemotherapy and easier to remove. As for marital status, married patients may receive spiritual and financial support from their families compared with unmarried patients (27), and thus choose more intensive treatment and have a better prognosis. Similar to previous studies, our study also found a survival advantage for women over men in stage IV pancreatic cancer (28). In summary, the model has good discriminating and calibrating capabilities to select patients with stage IV pancreatic cancer who could potentially benefit from pancreatectomy and chemotherapy.

This study also has certain limitations. Firstly, because our study is a retrospective study and patients with unclear clinicopathological information were deleted, there is a possibility of selection bias. Secondly, there is no key information in the SEER database including physical status, nutritional status, details of the surgery, chemotherapy regimens, chemotherapy course, chemotherapy and surgery sequence, etc., and sarcopenia has recently been recognized as a risk factor for postoperative pancreatic cancer (29). Therefore, the inclusion of these important factors may make our model more accurate. Finally, although our model has good performance in internal validation, we still need to evaluate the accuracy of the model based on external verification of independent cohorts. Nevertheless, considering the scale of our study and the rigorous statistical calculations, the conclusions of the study are still credible.

## Conclusion

In summary, this study used a large population-based SEER database to examine the influencing factors and the efficacy of surgery and chemotherapy in patients with mPC. We found that surgery and chemotherapy prolonged the overall survival of some mPC patients, and we established a nomogram to screen out those patients who might benefit. However, it is necessary to carefully evaluate the clinical effectiveness of pancreatectomy and chemotherapy in mPC. And further prospective studies are needed for verification.

## Data Availability Statement

Publicly available datasets were analyzed in this study. This data can be found here: https://seer.cancer.gov/.

## Author Contributions

JC, JL, and DN conceived and designed the study. DN and GL collected clinical data and performed the statistical analysis, GA, SL, and ZW prepared the figures and tables. All authors contributed to the article and approved the submitted version.

## Funding

This work was supported by a grant from the Key Research and Development Projects in Hunan Province (No.2018SK2127).

## Conflict of Interest

The authors declare that the research was conducted in the absence of any commercial or financial relationships that could be construed as a potential conflict of interest.

## Publisher’s Note

All claims expressed in this article are solely those of the authors and do not necessarily represent those of their affiliated organizations, or those of the publisher, the editors and the reviewers. Any product that may be evaluated in this article, or claim that may be made by its manufacturer, is not guaranteed or endorsed by the publisher.
